# The physiological origin of task-evoked systemic artefacts in functional near infrared spectroscopy

**DOI:** 10.1016/j.neuroimage.2012.02.074

**Published:** 2012-05-15

**Authors:** Evgeniya Kirilina, Alexander Jelzow, Angela Heine, Michael Niessing, Heidrun Wabnitz, Rüdiger Brühl, Bernd Ittermann, Arthur M. Jacobs, Ilias Tachtsidis

**Affiliations:** a Free Univ. Berlin, Habelschwerdter Allee 45, 14195 Berlin, Germany; b Physikalisch-Technische Bundesanstalt (PTB), Abbestr. 2–12, 10587 Berlin, Germany; c University College London, Dept. Med. Physics and Bioengineering, Gower Street, London WC1E 6BT, UK

**Keywords:** Time-domain optical brain imaging, Systemic changes, Skin blood flow, Periphery physiology, fMRI, Frontal lobe

## Abstract

A major methodological challenge of functional near-infrared spectroscopy (fNIRS) is its high sensitivity to haemodynamic fluctuations in the scalp. Superficial fluctuations contribute on the one hand to the physiological noise of fNIRS, impairing the signal-to-noise ratio, and may on the other hand be erroneously attributed to cerebral changes, leading to false positives in fNIRS experiments. Here we explore the localisation, time course and physiological origin of task-evoked superficial signals in fNIRS and present a method to separate them from cortical signals. We used complementary fNIRS, fMRI, MR-angiography and peripheral physiological measurements (blood pressure, heart rate, skin conductance and skin blood flow) to study activation in the frontal lobe during a continuous performance task. The General Linear Model (GLM) was applied to analyse the fNIRS data, which included an additional predictor to account for systemic changes in the skin.

We found that skin blood volume strongly depends on the cognitive state and that sources of task-evoked systemic signals in fNIRS are co-localized with veins draining the scalp. Task-evoked superficial artefacts were mainly observed in concentration changes of oxygenated haemoglobin and could be effectively separated from cerebral signals by GLM analysis. Based on temporal correlation of fNIRS and fMRI signals with peripheral physiological measurements we conclude that the physiological origin of the systemic artefact is a task-evoked sympathetic arterial vasoconstriction followed by a decrease in venous volume.

Since changes in sympathetic outflow accompany almost any cognitive and emotional process, we expect scalp vessel artefacts to be present in a wide range of fNIRS settings used in neurocognitive research. Therefore a careful separation of fNIRS signals originating from activated brain and from scalp is a necessary precondition for unbiased fNIRS brain activation maps.

## Highlights

► Task-evoked changes in the skin blood volume induce artefacts in fNIRS signals. ► Artefacts result from a venous volume decrease due to sympathetic vasoconstriction. ► This phenomenon might induce false positives in fNIRS experiments on the forehead.

## Introduction

Functional Near-Infrared Spectroscopy (fNIRS) is a non-invasive technique for studying the operative organization of the human brain by measuring haemodynamic responses to neuronal activation in the cerebral cortex. Due to its cost efficiency, its possibility for use at the bedside and compatibility with electrophysiological methods, fNIRS has a high potential as an imaging method for clinical studies, basic and applied research. Furthermore, it can substitute and complement functional Magnetic Resonance Imaging (fMRI) in a number of applications such as in developmental research (Gervain and Bortfeld, 2011; Lloyd-Fox et al., 2010) or when a combination with EEG or MEG is required.

A major challenge of fNIRS is its high sensitivity to haemodynamic fluctuations in the scalp (Gregg et al., 2010; Tachtsidis et al., 2009; Takahashi et al., 2011). The sensitivity of fNIRS to haemodynamic changes in superficial scalp layers was estimated to be 10 to 20 times higher than to changes in deeper cortical tissue, depending on the source–detector distance and the model used for the estimation (Al-Rawi et al., 2001; Liebert et al., 2004). In addition, when compared to the cerebral compartment, where the blood flow is stabilized by cerebral autoregulation, the blood flow in the skin is more strongly affected by changes in the systemic physiology. Therefore physiological noise induced by heart beat, breathing cycle, low frequency oscillations of blood pressure and heart rate around 0.1 Hz (Mayer waves) often account for more than 10% of fNIRS signal changes, leading to a lower sensitivity of fNIRS to cerebral activation as compared to fMRI (Boas et al., 2004; Tachtsidis et al., 2009).

Moreover, task-evoked changes in skin perfusion can mask changes in cerebral activation in fNIRS experiments. Such task-evoked changes result either from systemic blood pressure changes (Boas et al., 2004) or from skin-specific regulation mechanisms. Recently it was demonstrated that blood pressure fluctuations can exert confounding effects on brain fNIRS, through expression in extracranial tissues and within the brain itself (Minati et al., 2011). The skin blood flow is not only responsible for the metabolic supply of the skin, but also plays an important role in the regulation of local and global body temperature, fight-and-flight responses and stabilization of blood pressure in upright posture. These functions are maintained by the interplay of local control mechanisms and the input from the autonomic nervous system, which includes sympathetic and parasympathetic influences (Drummond, 1996). For example, the blood flow in the forehead is affected by a number of factors such as local and global body temperature, pain, gustatory stimulation, cognitive and social stress, and emotions (Drummond, 1997). Therefore, changes in the skin blood volume might be evoked by many experimental task paradigms currently used in fNIRS studies, in particular in complex settings involving emotional and cognitive processing. Since these changes are stimulus-locked and hence cannot be separated from cortical signals by appropriate filtering, they might produce false positives in fNIRS activation maps (Tachtsidis et al., 2009; Takahashi et al., 2011).

Several methods were proposed to separate cerebral and extracranial signals in fNIRS. In a part of these methods additional measurements of superficial haemodynamics are performed which are then used to eliminate the extracranial contribution in the fNIRS signals. Extracranial haemodynamic can be estimated either by means of an additional short-distance detector (Choi et al., 2002; Saager and Berger, 2005; Saager et al., 2011), NIRS tomography (Gregg et al., 2010) or analysis of the photon time-of-flight distribution in time-domain NIRS (Aletti et al., 2012; Liebert et al., 2004). Most of these methods rely on the assumption of a homogeneous spatial distribution of extracranial signals across the scalp. This assumption may be justified for haemodynamic fluctuations in capillaries or small vessels, but may be less accurate for the contribution of big arteries and veins supplying the scalp (Gagnon et al., 2012).

Another group of methods explores differences in the temporal evolution of the extra- and intracerebral haemodynamic signals. Adaptive filtering (Zhang et al., 2009), statistical parametric mapping (SPM) where systemic changes were included as regressors into the model (Tachtsidis et al., 2010), independent component analysis (Kohno et al., 2007; Patel et al., 2011) and superficial signal regression assume statistical independence of superficial and cerebral signals. However, statistical independence might be violated if skin perfusion changes are induced by the task.

To justify the above-mentioned assumptions, a thorough understanding of the physiology underlying the superficial fNIRS signals is necessary. The aim of the present study is to explore the anatomical localisation, temporal characteristics and physiological origin of superficial signals in fNIRS and to develop a method to separate them from cortical signals.

To achieve these aims, we used a multimodal approach combining time-domain fNIRS with fMRI and simultaneous measurement of peripheral physiological parameters. Time-domain fNIRS provides a possibility to separate extracranial and cerebral haemodynamic signals by measuring the photon time-of-flight distribution. fMRI data obtained on the same task and the same subject population were used in order to localize the true cerebral activation with respect to the fNIRS optodes. In addition, we exploit the ability of fMRI to detect and localize haemodynamic changes in the skin. This is a novel approach, since information about skin haemodynamic changes is neglected in most fMRI and fMRI/fNIRS studies due to the brain masking during the fMRI data analysis. In order to understand the physiological origin of task-evoked extracranial signals, we complement the fNIRS measurement with measurements of peripheral physiology. To account for multiple possible regulation mechanisms of skin blood flow we measured global blood circulation parameters of heart rate and blood pressure and assessed local circulation in the forehead skin by means of laser Doppler flowmetry and the response of the autonomic nervous system by recording skin conductance.

As a region of interest we chose the prefrontal cortex, i.e. Brodmann area 10 (BA 10). This area is involved in important processes such as decision making, managing of subgoals (Braver and Bongiolatti, 2002) and empathy processing, to name just a few (Kringelbach, 2005; Gilbert et al., 2006). It is easily accessible for fNIRS and was investigated in a number of fNIRS studies on infants and adults (Schroeter et al., 2004). In addition, we expect blood volume fluctuations in the forehead in response to cognitive and emotional processing to be especially pronounced, since the forehead is involved in the phenomenon of social blushing (Drummond, 1997). Indeed, several studies reported stimulus evoked systemic artefacts in fNIRS of the forehead (Tachtsidis et al., 2009; Takahashi et al., 2011). For example, in a recent study Takahashi et al. (2011) used a combination of fNIRS and laser Doppler flowmetry to demonstrate that a substantial fraction of the haemodynamic response in the forehead originates from the cutaneous vascular response to a verbal fluency task.

## Methods

### Subjects

Fifteen healthy subjects (5 female/10 male, mean age 34.9 ±7.2 years, 13 right-handed, 2 left-handed) participated in two subsequent fNIRS and fMRI experiments performing a semantic Continuous Performance Task (sem-CPT). Thirteen subjects first took part in the fNIRS experiment, and subsequently, 1 h later, in the fMRI experiment. For one subject the fNIRS and fMRI sessions were separated by 1 week, and one subject took part first in the fMRI and 1 week later in the fNIRS experiments. Five subjects participated in an additional high-resolution fMRI session, which was scheduled 2 to 4 weeks after the main experiment. In this session we used a different task (n-back working memory task), (i) in order to avoid training and habituation effects due to the two previous sessions, (ii) to keep the high-resolution fMRI experiment comparable to the previous sessions with respect to the task-evoked autonomic arousal.

Thirteen subjects were German native speakers; two subjects were fluent in German. All participants gave informed consent in compliance with a protocol approved by the local Ethics Committee. None of the participants reported a history of neurological diseases. One subject reported problems with high and another one with low blood pressure, but none received pharmacological treatment.

### Tasks

#### Semantic Continuous Performance Task

In order to trigger activation in BA10, we used a variation of the Continuous Performance Task (CPT) (Braver and Bongiolatti, 2002) that included a semantic categorization task. A list of German words was presented to the subjects using rapid serial visual presentation (RSVP) with word presentation time of 1 s and 1 s inter-stimulus interval. Each of the words represented either a concrete or an abstract concept. In the semantic-CPT condition (sem-CPT) the subjects were instructed to press the left button whenever they saw a concrete word that followed an abstract one. The right button had to be pressed in any other case. In a control task (word-CPT) the subjects were instructed to press the left button every time they saw the word ‘KOFFER’ (suitcase), but only when it followed the word ‘VORZUG’ (preference).

The experiment consisted of 18 activation blocks (9 word-CPT blocks, and 9 sem-CPT) with a duration of 34.15 s each, separated by 31.15 s resting intervals during which a black screen with a fixation cross was presented. A 500 ms instruction screen (KOFFER<>VORZUG for word-CPT and ABSTRACT<>CONCRETE for sem-CPT) was presented before each block. In the fMRI experiment, the 18 blocks were divided into 3 runs with 6 blocks each. In the fNIRS experiment, the subjects performed 18 blocks in one run, starting with 120 s of a pre-run baseline, and ending with 120 s of post-run baseline.

#### N-back task

Five subjects participated in an additional high resolution fMRI experiment performing n-back task (Kirchner, 1958). Series of numbers from 1 to 4 were presented to the subject. During the 2-back blocks, subjects were instructed to press the button when the number shown on the screen was equal to the number presented two steps before. During the control blocks, subjects were instructed to press the button for each number. For both conditions, the blocks had a duration of 30 s. The experiment contained 15 stimulation blocks.

#### Stimulus presentation

The stimulation paradigms were implemented in the software package Presentation (Version 7, Neurobehavioral Systems).

During the fMRI, subjects were lying in the MR-scanner and the experimental stimuli were presented through a 90° mirror fixed to the MR receiver head coil. A fibre optic response device (4 Button Diamond, fORP-905, Current Designs Inc., Philadelphia, USA) was used to register the subjects' responses.

During the fNIRS experiment, the subjects were seated in front of standard 19-inch monitor (distance: 50 cm to 80 cm), on which the stimuli were presented. The responses were registered by a standard computer keyboard.

### Data acquisition and pre-processing

#### fMRI

Standard fMRI protocols employing an echo-planar imaging gradient echo pulse sequence were used for measurements of haemodynamic changes in the cerebral and extracranial compartments. The changes of the fMRI signal as measured with the gradient echo sequence reflect changes in the tissue blood volume and blood oxygenation. The fMRI signals from the brain recorded at 3 T mostly reflect tissue concentration of deoxy-haemoglobin due to the Blood Oxygenation Level Dependency (BOLD) effect (Buxton, 2002). The fMRI signals of the skin, which are usually neglected in the standard fMRI data analysis, but were analysed in the current study, are additionally influenced by the skin blood volume. The physical mechanism underlying the skin fMRI signals and their physiological interpretation are discussed in more detail in the Discussion section and in Appendix 2.

fMRI measurements were performed on a 3 T Verio (Siemens, Erlangen) scanner, equipped with a 32-channel coil. The gradient echo sequence (Echo-Planar-Imaging) was used (*T*_E_/*T*_R_/flip angle/bandwidth = 30 ms/2000 ms/70°/1689 Hz) with a 3 mm × 3 mm × 3 mm resolution, fat saturation prior to every slice and a GRAPPA acceleration factor of 2. Thirty-two nearly axially oriented slices with an interslice gap of 0.6 mm were acquired in an interleaved order, providing whole brain coverage.

Additional higher-resolution runs covering only the forehead (resolution 1.5 mm × 1.5 mm × 1.5 mm, 15 slices, *T*_E_/*T*_R_ = 30 ms/2000 ms) were performed for five subjects during their performance of the n-back task.

*T*_1w_ anatomical images (MPRAGE *T*_E_/*T*_R_/*T*_I_/flip angle/bandwidth = 3.03 ms/2300 ms/900 ms/9°/130 Hz, 1 mm × 1 mm × 1 mm resolution) and whole-head phase-contrast angiography (slice thickness 0.9 mm, in-plane resolution 0.41 mm × 0.41 mm, *T*_E_/*T*_R_ = 6.85/43.7 ms, flip angle = 15°, flow encoding velocity = 20 mm/s, 3D flow direction) were acquired for each subject. Fiducial markers (BrainLAB AG, Germany) were placed at the positions of the fNIRS optodes.

fMRI data analysis was performed using the Brain Voyager QX software (Version 2.0, Brain Innovation, Netherlands). Pre-processing included motion correction, temporal detrending, slice time correction and co-registration with anatomical *T*_1w_ images. One subject was excluded from the fMRI analyses due to pronounced motion artefacts.

Two types of analyses were performed separately, an analysis of cerebral activation and an analysis of extracranial signals.

##### Cerebral activation analysis

For the analysis of cerebral activation, images were normalized into standard Talairach space. The design matrix for the analysis of cerebral activation included two predictors, which represented boxcar functions corresponding to onsets of sem-CPT and word-CPT blocks convolved with the haemodynamic response function (HRF). The default HRF of Brain Voyager was used (two Gamma HRF, onset of response and undershoot 5 s and 16 s, respectively, dispersion 1 s, response to undershot ratio 6).

For the cerebral activation, second level analysis was performed on the contrast beta-maps of single subjects. An average anatomical template was constructed by averaging the *T*_1w_ images across the subject population in Talairach space.

##### Extracranial signals analysis

Since normalization into standard space does not provide co-alignment of the scalp, the analysis of extracranial fMRI signals was performed on the single-subject level only. For this purpose fMRI images were co-registered with *T*_1w_ anatomical images and MR-angiography of each subject. The design matrix for the analysis of extracranial signals included two predictors corresponding to sem-CPT and word-CPT tasks, and an additional predictor simulating the time course of extracranial task-evoked signals, which is described in the following section.

#### fNIRS

The concentration changes in oxy- and deoxy-haemoglobin (Oxy-Hb and Deoxy-Hb, respectively) on the forehead (BA10) were measured using the PTB time-domain brain imager (Wabnitz et al., 2005). The device is equipped with picosecond diode lasers (Sepia, Picoquant, Germany) of three wavelengths (689 nm, 797 nm, 828 nm) operated at a repetition rate of 41.9 MHz. The light of all three lasers was combined and equally split into two source fibres each providing the overall average power of 3 mW at the output end. The diffusely reflected light was collected by four detection fibre bundles (diameter 4 mm, numerical aperture NA 0.54, length 1.4 m, Loptek Glasfasertechnik, Germany). The time-resolved detection was performed by fast photomultipliers connected to a multi-board time-correlated single photon counting (TCSPC) system (SPC-134, Becker & Hickl GmbH, Germany). Distributions of times of flight of photons (DTOFs) were acquired with a temporal resolution of 24.4 ps and at a sampling rate of 20 Hz. The instrumental response function (IRF) was measured after each functional measurement with the procedure described in Liebert et al. (2003).

**Fig. 1:**
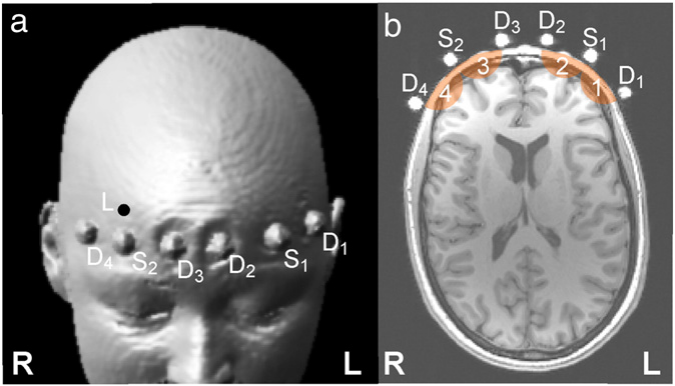
Positions of the fNIRS light sources (S_1_, S_2_), detectors (D_1_ to D_4_) and Laser Doppler Monitor probe (L) on a subject's forehead. (a) Surface rendering of the MR anatomical image; (b) axial slice through the MR anatomical image. The volume sampled by the corresponding fNIRS channels (1 to 4) is indicated in colour.

One source fibre and two detection fibre bundles were placed on each hemisphere with a source-detector separation of 3 cm. A source-detector separation of 3 cm provides a trade-off between sufficient light penetration depth on the one side, and a sufficient total number of photons to achieve a good signal-to-noise ratio in the time-domain fNIRS measurements. The optodes were inserted into the holes of a foam rubber holding band, which was tightly fixed on the subject's head with a Velcro strap. In the following, we refer to the sampled volume of the four source-detector pairs as channels 1 to 4. The positions of light sources, detectors and corresponding fNIRS channels are illustrated in Fig. 1.

The pre-processing and GLM analysis of fNIRS data was performed in MATLAB (Mathworks Inc.).

In order to exploit the potential of time-domain fNIRS to separate extracranial and cerebral signals we analysed the time-resolved optical data in terms of statistical moments of DTOFs (Liebert et al., 2004). The time courses of the Oxy-Hb and Deoxy-Hb concentration changes were obtained from light attenuation changes derived from the total photon count *N*_0_ and additionally from changes in variance *V* of the measured DTOFs. The detailed procedure is described in Appendix 1. Note here that the haemoglobin concentration changes based on attenuation changes are analogous to the signals usually measured in continuous wave (cw) fNIRS experiments. The Oxy-Hb and Deoxy-Hb concentration changes obtained from the variance signal have the advantage to be less sensitive to superficial absorption changes (Liebert et al., 2004).

The time series were visually inspected to detect motional artefacts. Step-like signal changes attributed to movements were detected in the data of two subjects. These two subjects were excluded from further analysis. The time series of concentration changes were subjected to temporal filtering. We applied two filters (zero phase, 5th order Butterworth filters, MATLAB Signal Processing Toolbox) to all haemoglobin time series data, (i) a low-pass filter with a cut-off frequency of 0.5 Hz to suppress heart beat and high frequency noise, (ii) a high-pass filter with a very low cut-off frequency of 0.001 Hz to remove long-time drifts. For the block averaging analysis, each block was re-baselined to the mean value in the interval between 7 s and 3 s prior to the stimulation onset.

#### Peripheral physiology

During the fMRI experiments the subject's heart rate was recorded by the scanner's integrated pulse plethysmograph placed on the left-hand forefinger.

During the fNIRS experiments a number of systemic parameters were recorded simultaneously, i.e. heart rate (from electrocardiography), blood pressure (BP), skin conductance (SC), scalp blood flow (SBF) and scalp concentration changes of red blood cells (RBC). Electrocardiography (ECG) and SC were recorded using a Nexus-10 (Mind Media). SC was measured between the middle and the ring fingers of the left hand. The ECG electrode placement on the chest was: ground — upper left part, negative — upper right part, positive — lower left part of the chest. Mean blood pressure was measured at the index finger of the left hand by the PortaPress system (TNO TPD Biomedical Instrumentation). Changes in scalp blood flow (flux) and concentration of red blood cells were recorded by the floLAB Laser Doppler Perfusion Monitor (Moor Instruments). The laser Doppler instrument operates at 670 nm (red wavelength) and a very short emitter detector distance (~ 1 mm); hence the penetration depth is between 550 and 750 μm. Its probe was placed on the right forehead over one of the tdNIRS sources (S2). A low-pass filter with a cut-off frequency of 0.8 Hz was applied to the RBC and SBF signals in order to remove the heart beat. The other signals did not show influence of the cardiac pulse (i.e. SC, BP).

## Results

### fMRI: cerebral activation

**Fig. 2:**
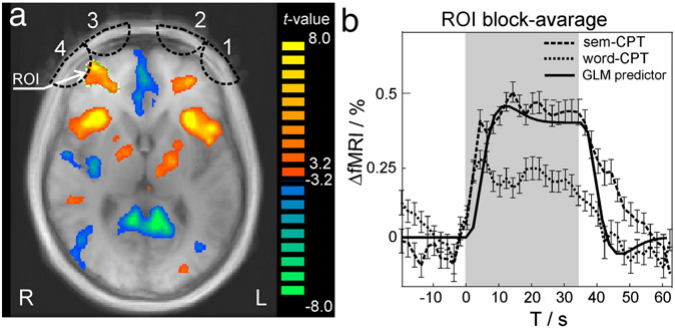
Results of the group analysis of cerebral activation measured with fMRI. (a) Activation map for the group analysis of fMRI data overlaid on the axial slice of the group anatomic template for contrast (sem-CPT + word-CPT) vs. (fixation cross baseline), (p(Bonf) < 1.000, p < 0.007) The activation is observed in the right Brodmann Area 10 underneath fNIRS channels 3 and 4 (centre at Talairach coordinates (32, 48, 11), volume of 7665 mm^3^). (b) Block average of the fMRI signal time courses in the activated region of interest (ROI in right Brodmann Area 10) for both tasks overlaid with the GLM predictor used to model cerebral activation.

Fig. 2a shows the activation map for the contrast of sem-CPT + word-CPT vs. fixation cross baseline from the second level group analysis of the fMRI data overlaid on a group anatomical template. The list of regions activated in this contrast is summarized in Table 1 of the Supplementary material. In the following analysis we will focus on the prefrontal cortex, since only this area was probed by the four fNIRS channels. Two regions of the prefrontal cortex were activated in both tasks. We found bilateral activation in BA10 corresponding to fNIRS channel positions 1 and 4 with maxima at Talairach coordinates (32, 48, 11) and (− 29, 42, 14), with a lateralization to the right. Both regions were more strongly activated by the sem-CPT than by the word-CPT condition. The time course of the fMRI signal in the activated area (activated ROI defined by threshold of t > 3.3) averaged over all subjects and all block-repetitions is shown in Fig. 2b. The shape of the haemodynamic response follows the pattern typically found for cerebral activation. The signal intensity increases with the onset of the task, reaching the plateau about 10 s after the task, and decays back to the baseline after the end of the task period.

Deactivation was observed in the medial regions, Talairach coordinates (3 56 13), with no significant difference between the two tasks.

### fMRI: extracranial signals

**Fig. 3:**
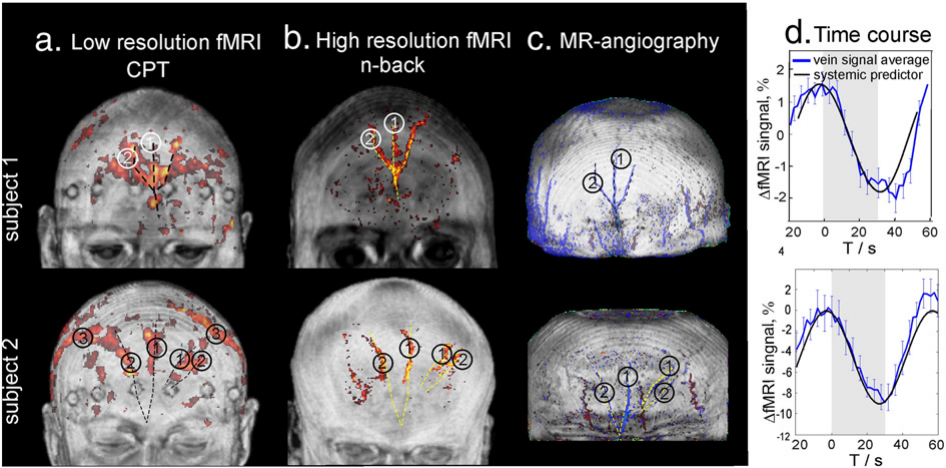
Results of extracranial signals measured with fMRI for two representative subjects (row 1 and row 2). (a) Extracranial activation map during a CPT task measured by a whole head fMRI with a low resolution (3 × 3 × 3 mm^3^); (b) extracranial fMRI activation during an n-back task measured by fMRI of the forehead with high resolution (1.5 × 1.5 × 1.5 mm^3^); (c) MR angiography. The veins are indicated in blue, and arteries in red. The numbers indicate the position of (1) frontal (supratracheal) (2) supraorbital and (3) supratemporal veins; (d) Time course of fMRI signal averaged over all repetitions of the CPT-task and over all voxels containing superficial frontal veins.

In the CPT experiment, the amplitudes of fMRI signal fluctuations in the scalp were analysed on the single subject level. Strong task-evoked variation of the MR signal was found for a series of contiguous voxels in the scalp. The extracranial activation maps overlaid on surface reconstructions from anatomical images for two representative subjects are shown in the first column of Fig. 3a. Periodic signal changes with a period of a single block were observed for all subjects. Block averages of the signal across the voxels with strong stimulus-evoked signal modulations are presented in Fig. 3d. The signal decreases continuously during the stimulation block and continuously returns to its initial value during the rest period. As can be seen in Fig. 3, amplitudes of the fluctuation and patterns of voxels vary strongly between the subjects. The scalp activation patterns and the time course of the block average were reproducible between runs for each subject.

Similar task-evoked fluctuations in the scalp were observed for the high resolution forehead fMRI images obtained during the n-back task performance (see Fig. 3b). Comparison of Figs. 3a and b shows that the scalp activation revealed spatial patterns similar to those observed for the CPT task, provided that the different spatial resolution of both measurements is taken into account.

A GLM analysis of the extracranial signals was performed for both, the CPT (low resolution fMRI) and the n-back (high resolution fMRI) experiments. In addition to the two predictors modelling brain activation in the word-CPT and sem-CPT conditions (boxcar convolved with HRF function), we used a periodical predictor to model the extracranial signals. In particular, a cosine function with the period of a single block and the phase locked to the task onset was used (see Fig. 3d). The resulting activation maps are shown in Figs. 3a and b.

Fig. 3c shows the angiography of the superficial vessels for each subject overlaid on the surface reconstruction of the anatomical MR images. Comparison of the positions and topologies of vessels draining the scalp (see Fig. 3d) with the activation pattern in Figs. 3a and b reveals that activated voxels are co-localized with the veins draining the scalp. Task-evoked fMRI signal changes were observed for all three pairs of veins draining the forehead, i.e. supraorbital veins, frontal (supratrochlear) veins and superficial temporal veins. According to the angiographic image the veins exhibiting the task-evoked signal changes have a diameter of about 1 mm to 2 mm, and are located in the hypodermis 3 mm to 5 mm below the skin surface.

### fNIRS

**Fig. 4:**
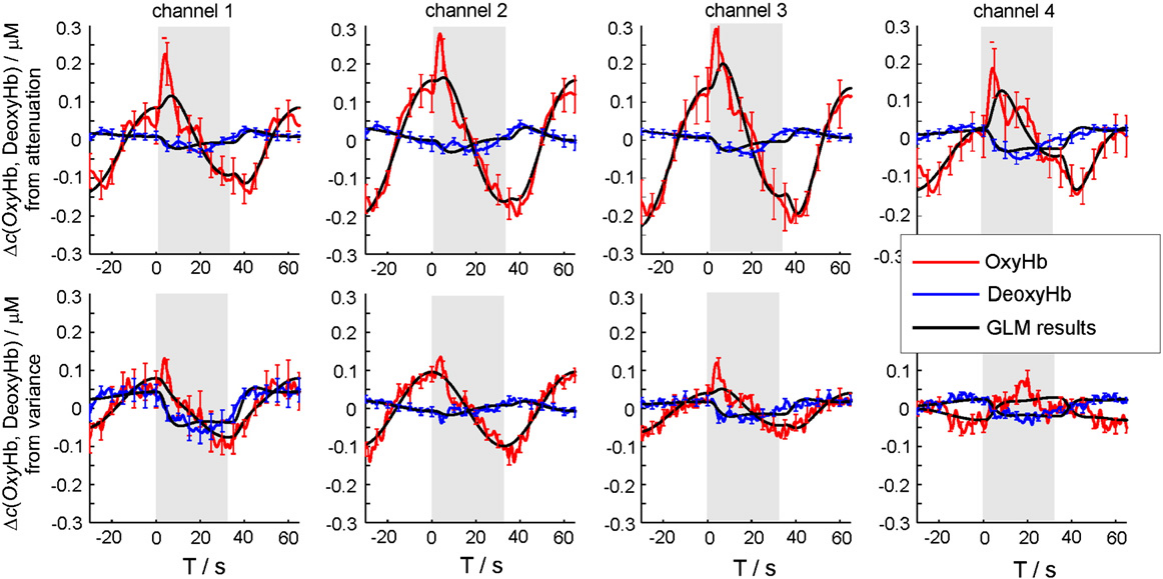
Results of the group average of fNIRS data. Block average of fNIRS signals over all subjects and all task blocks of both word-CPT and sem-CPT tasks overlaid with the best fit from the General Linear Model analysis is shown. From left to right: channels 1 to 4. Top row: signals extracted from the light attenuation (analogues to conventional cw-fNIRS signals). Bottom row: signals extracted from variance *V* of DTOFs. These signals have a smaller contribution of absorption changes in skin layers as compared to cw-fNIRS signals. The periods of the task are indicated as grey blocks. The error bars indicate the standard error of mean.

The Oxy-Hb and Deoxy-Hb concentration changes for four fNIRS channels averaged over all subjects and all repetitions of the task blocks are shown in Fig. 4. The top row in Fig. 4 corresponds to the concentration changes of Oxy-Hb and Deoxy-Hb obtained from the changes in light attenuation. The bottom row corresponds to the Oxy-Hb and Deoxy-Hb concentration changes estimated from the changes in variance of the DTOFs. Strong task-evoked changes were observed for all four channels, two haemoglobin species, and attenuation as well as variance based signals. However, the shape of the response is different from what was expected for cerebral activation based on the literature on task-related brain activation and on the results of the analysis of cerebral fMRI signals from the current study (see Fig. 2b). Periodic changes similar to those observed in the scalp-fMRI (see Fig. 3d) dominate the changes of the Oxy-Hb group average signals. The amplitude of these signals is higher for the two medial channels compared to the lateral channels, and is more pronounced in the Oxy-Hb signal than in the Deoxy-Hb signal.

A GLM analysis was performed on the fNIRS data. The model included a cerebral predictor modelling responses to sem-CPT and word-CPT blocks (see Fig. 2b) and an additional extracranial cosine predictor to model task-evoked scalp haemodynamic changes. The time course of extracranial predictor was adopted from the analysis of the fMRI signals of cutaneous veins (see Fig. 3d). As in the GLM analysis of fMRI data, the phase of the cosine predictor was fixed with respect to the onset of the stimulation blocks.

The best fit obtained by the GLM model averaged across all subjects and block repetitions overlaid on the group and block averaged of the data is shown in Fig. 4.

**Fig. 5:**
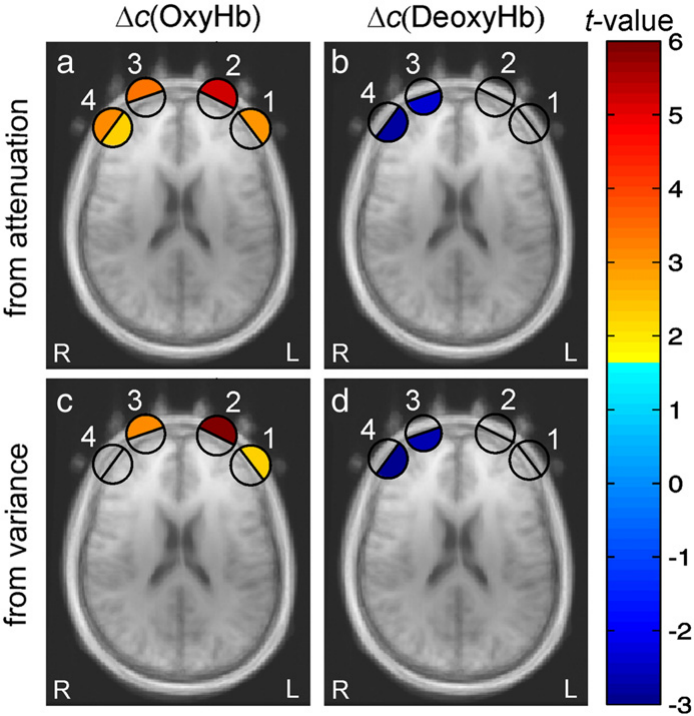
*t*-Value activation maps (oxy/de-oxy, intra/extracranial) derived from the GLM group analysis of fNIRS data overlaid on anatomical MR images. The coloured half circles represent the *t*-values attributed to the Oxy-Hb (a, c) and Deoxy-Hb (b, d), intra- (inner part of the circle) and extracranial (outer part of the circle) predictors for those channels which show a signal with a significance level p < 0.05. Empty circles correspond to non-significant t-values. Top row (a, b): signals extracted from the light attenuation signal (analogues to conventional cw-fNIRS signals). Bottom row (c, d): signals extracted from the variance of DTOFs. The corresponding concentration changes averaged over the subject population are shown in Table 1.

As a result of the single subject GLM analysis, we obtained *β*-values (predictor coefficients) for the amplitudes of extracranial and cerebral predictors for each of the 14 subjects, 4 NIRS channels, two haemoglobin species, light attenuation and variance based signals. The obtained *β*-values were subjected to the second level group analysis by performing one-sample *t*-tests across the subject population. The resulting *t*-values are presented in Fig. 5 in colour code. The amplitudes of the group averages of concentration changes are summarized in Table 1. A significant cerebral activation was found for channel 4 for Oxy-Hb, and for channels 3 and 4 for Deoxy-Hb for the contrast (sem-CPT + word-CPT) vs. baseline. No significant differences were found between sem-CPT and word-CPT. The significant extracranial signal was present in all channels for Oxy-Hb based on attenuation and for channels 1, 2 and 3 for variance based signals, with higher amplitudes in both medial channels. No significant extracranial signals were found for Deoxy-Hb.

### Peripheral physiological measurements

**Fig. 6:**
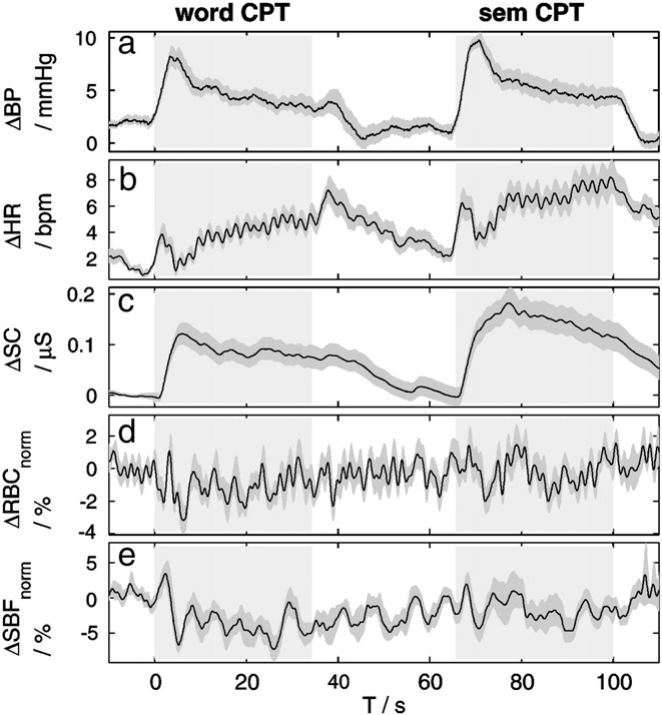
Task-evoked changes in (a) blood pressure, (b) heart rate, (c) skin conductance, (d) red blood cell concentration changes and (e) scalp blood flow measured by Laser Doppler. The time traces are averaged over all subjects and all block repetitions. The periods of the word-CPT and sem-CPT tasks are indicated with grey blocks. The grey bands indicate the standard error of mean.

The time courses of all physiological measurements recorded during the fNIRS experiment averaged over subjects and block repetitions are presented in Fig. 6. The task-evoked changes in the SC indicate elevated sympathetic outflow during task performance. The heart rate and blood pressure exhibit task-evoked changes, too. The blood pressure increases during the task performance and returns back to baseline during the rest period. The initial increase and immediate decrease of the heart rate with the onset of the task is followed by a monotonous increase during the task block. During the rest period the heart rate is monotonously decreasing and returns to baseline.

The measurements of task-evoked heart rate changes performed during the fMRI experiment show comparable results (data not shown).

The red blood cell concentration in the skin measured by Laser Doppler does not show any significant changes during the task performance, while skin blood flow reveals an average decrease of 4% during the performance of the CPT task.

## Discussion

We observed a strong task-evoked systemic component in the fNIRS signals measured on the forehead during the performance of a cognitive task (see Fig. 4). This component was identified as a systemic artefact based on its time course which differs from that of a typical task-evoked cerebral haemodynamic response. In addition, in contrast to cerebral activation signals, the artefacts were observed for Oxy-Hb and total Hb only, but not for Deoxy-Hb concentration changes. Similar task-evoked changes were observed in the fMRI signals from cutaneous veins draining the forehead (see Fig. 3d). Based on the striking similarity of the signal time courses of the two modalities, we assume their common localization and related physiological origin. Thereby we conclude that the main source of artefacts in fNIRS is task-evoked haemodynamics of the cutaneous veins.

In the following we will outline these findings in more detail based on the different physical observables that are measured by fNIRS and fMRI, respectively. We will then bring these strands of research together with peripheral physiological measurements to better understand the origin of fNIRS artefacts, the main objective of the present study.

### Extracranial fNIRS and fMRI signals

The amplitude of the single voxel MR-signal *S* obtained with the gradient echo sequence with evolution time *T*_*E*_ is determined by tissue water content *M*_*v*_ and tissue dephasing time *T*_2_*.

(1) $$S\sim M_{v}*\exp\left(-\frac{T_{E}}{T_{{}_{2}}^{*}}\right)$$

Therefore task-evoked changes of fMRI signals can originate from changes of either parameter. Regarding fMRI signals from the brain, it was demonstrated theoretically and experimentally that Echo-Planar-Imaging at 3 T reflects mostly changes in the tissue dephasing time *T*_2_* induced by changes in the local concentration of the paramagnetic Deoxy-Hb. Due to this reason the majority of combined fMRI/fNIRS studies found that cerebral fMRI signals are negatively correlated with Deoxy-Hb and positively correlated with Oxy-Hb concentration changes (Steinbrink et al., 2006) with Deoxy-Hb showing higher absolute correlation values. A comparison of the brain activation map obtained with fMRI (Fig. 2) with the cerebral activation from fNIRS (inner halfcircles in Fig. 5) demonstrates a similar trend.

In contrast to the brain signals, the skin fMRI signals are strongly influenced by water and blood content in a voxel. In our study the extracranial signal from fMRI was found to be highly correlated with the Oxy-Hb concentration changes, and does not reveal a significant contribution from Deoxy-Hb (compare the skin fMRI activation map in Fig. 3 with the outer halfcircles in Fig. 5). To understand this difference between cerebral and extracranial fMRI signals one has to realize that the theoretical framework used to interpret cerebral fMRI signals has to be modified when fMRI signals from the skin are considered. The peculiarity of skin structure and its MR properties are discussed in more detail below and a theoretical model for the interpretation of cutaneous fMRI signals is developed.

Typically skin tissue exhibits low fMRI signals due to its low water content and short relaxation time *T*_2_. The outer skin layers, epidermis (thickness about 100 μm) and dermis (up to several mm thick), together with two corresponding vascular plexuses (vessel diameter of 20 μm to 200 μm) appear as an unresolved single layer in fMRI images (Bittoun et al., 2006). The third layer, hypodermis, with a thickness of up to 1 cm, contains mostly fatty tissue and larger blood vessels (1 mm to 2 mm diameter). Due to its low water content hypodermal tissue appears dark in conventional fMRI when fat suppression is applied (Laistler et al., 2011). Big veins, however, with millimetre diameters yield comparatively strong fMRI signals despite the strong partial volume effects.

We observed the task-evoked changes in the fMRI signal only for those voxels in the hypodermis that contain big cutaneous veins, and not for dermal layers with smaller vessels. For a correct interpretation of these signals one has to note that the extra-vascular compartment contributes differently to the fMRI signals of the brain and the skin. In contrast to the brain where extravascular tissue is responsible for more than 50% of the fMRI signal at 3 T (Uludag et al., 2009), the extravascular contribution to the fMRI signal from the skin is negligible due to an intrinsically low tissue signal. Therefore the task-evoked decrease of the cutaneous venous fMRI signal, observed in our experiments, can clearly be attributed to intravascular changes. The underlying mechanism is either a decreased venous blood oxygenation and the related shortening of blood relaxation time *T*_2_ or a decreased venous water volume or a combination of both.

The relative contribution of either mechanism can be estimated based on a water relaxation model (Uludag et al., 2009), resulting in

(2) $$\frac{\text{Δ}S}{S}\approx 1.6\frac{\text{ΔΣ}\left(\text{OxyHb}\right)}{\text{Σ}\left(\text{OxyHb}\right)+\Sigma \left(\text{DeoxyHb}\right)}-0.4\frac{\text{ΔΣ}\left(\text{DeoxyHb}\right)}{\text{Σ}(\text{OxyHb})+\text{Σ}(\text{DeoxyHb})}$$

where *S* denotes total MRI signal from the vein, Σ (Oxy-Hb) and Σ (Deoxy-Hb) denote the baseline total amount of oxy- and deoxy-haemoglobin, respectively, in the cutaneous veins while the Δ*S* and ΔΣ terms in the numerators represent the task-related changes of these quantities. The derivation of Eq. (1) and details of the underlying assumptions are given in the Appendix 2. Note that the fMRI signal changes contain a positive contribution from the changes of Oxy-Hb with a weighting factor of 1.6, while changes of Deoxy-Hb have a negative contribution with a weighting factor being four times smaller. By analysis of skin fMRI data alone the relative contributions of Oxy-Hb and Deoxy-Hb cannot be separated. Further insight may be gained by complementing the fMRI data by fNIRS data which allows for independent measurements of the Oxy-Hb and Deoxy-Hb concentrations in the tissue.

Strong task-evoked changes with a time course similar to that observed for fMRI signals of cutaneous veins were found in the fNIRS data of all subjects (compare Figs. 3d and 4). These signals were observed for Oxy-Hb but not for Deoxy-Hb concentration changes and had higher amplitudes in the two medial channels (see Fig. 5). The average amplitude of the extracranial predictor contribution obtained for the attenuation-based signals was found to be at least twice as high as the one obtained from the variance-based signals (see Table 1). This finding together with the different depth selectivity of attenuation and variance supports the assumption of the superficial localisation of signals under discussion.

In combination with the skin fMRI signals these results shed light on possible mechanisms of task-evoked haemodynamics of the skin. Note that the changes of the average tissue concentrations Δ*c*(Oxy-Hb) and Δ*c*(Deoxy-Hb) as measured by fNIRS are proportional to the changes of the total amount of ΔΣ(Oxy-Hb) and ΔΣ(Deoxy-Hb) in the cutaneous veins which affect the fMRI signal according to Eq. (1). The fNIRS data of Fig. 4 and Table 1 indicate that no significant changes of the extracranial Deoxy-Hb concentration occur, i.e. Δ*c*(Deoxy-Hb) ≈ 0. Thus the relative changes in the venous volume are given by Δ*c*(Oxy-Hb) / (*c*(Oxy-Hb) + *c*(Deoxy-Hb)). The second term of Eq. (1) can hence be neglected and we obtain the simple result that (*i*) the fMRI signal change is directly proportional to the relative change of the venous volume, and (*ii*) the scaling factor is 1.6. The two subjects presented in Fig. 3, for example, exhibit fMRI signal changes of 4% and 9%, corresponding to venous volume changes of about 2.5% and 5.5%, respectively.

The considerations presented above implicitly assume that task-evoked extracranial signals in fNIRS and fMRI originate from the same vascular compartment. Let us critically discuss this assumption taking into account that fNIRS and fMRI have different sensitivity to vessels of different sizes. As discussed above, skin fMRI was observed in veins with a diameter of 1.2 mm to 2 mm. The fact that superficial signals in fMRI were observed in big veins only does not necessarily mean that task-evoked haemodynamic changes in the skin are restricted to them. Arteries generally contain less blood and have smaller diameters. Therefore they are not visible in the fMRI images of the skin. The haemodynamic changes in smaller vessels of the dermis and the epidermis might not be visible due to the low partial volume of blood and short relaxation time in these compartments.

fNIRS, in contrast, is generally less sensitive to big vessels due to the high absorption and low probability of photon escape (van Veen et al., 2002). According to Liu et al. (Liu et al., 1995) the haemoglobin distributed in vessels of a diameter less than 0.7 mm has a maximal contribution to light absorption. However, these authors showed that vessels of 1.6 mm diameter still have a substantial effect on the tissue absorbance, albeit their contribution is underestimated by about 30%. Thus the haemodynamics of these vessels *will* contribute to fNIRS changes, in particular when the vessels are situated superficially in proximate vicinity to light source or detector.

The finding that superficial effects in fNIRS were strongest for the medial channels 2 and 3 is also in agreement with the fMRI results. Apart from a substantial inter-subject variability of the venous geometry, the supraorbital and supratrochlear veins are frequently joining at the root of the nose, i.e. beneath the position of the medial fNIRS channels 2 and 3. Based on these considerations we conclude that the decrease in fMRI skin signal and the task-evoked decrease in the concentration of oxygenated haemoglobin can be explained by the same physiological phenomena of haemodynamic in big cutaneous veins.

Beyond that, it is possible that fNIRS signals contain an additional contribution of small vessels in superficial dermal plexuses, which are invisible in fMRI.

In principle, this contribution could be assessed by considering the red blood cell concentration (RBC) measured with laser Doppler (see Fig. 6d). The RBC signal is expected to reflect systemic changes in total haemoglobin concentration in a near-surface compartment of the skin, i.e. within its penetration depth of 550 to 750 μm. It should be kept in mind that the RBC signal cannot contain contributions from the deeper lying veins (3 to 5 mm deep) that were discussed before as the major source of systemic fNIRS signals.

The RBC measurements did not reveal significant task-evoked changes similar to those observed by fNIRS. This means that even if the small task-evoked changes are present in the superficial vessel plexus it is hidden in the substantial noise of about 2% (see Fig. 6d). In order to relate this value to fNIRS results quantitative comparison of fNIRS and RBC signals have to be performed which has to take into account the different quantities measured. A 2% change in total haemoglobin concentration would correspond to about 2 μM haemoglobin concentration changes in the superficial layer, assuming a blood content of 5% in skin and a typical Hb concentration in blood of 150 g/l. Such a concentration change, restricted to a superficial skin layer, would lead to an fNIRS signal that is about an order of magnitude smaller due to a partial volume effect, i.e. the ratio between the partial optical pathlength in a thin superficial layer and the mean total pathlength (Liebert et al., 2004). This means that even small task-evoked changes hidden in noise of RBC measurement could evoke the effects in fNIRS measurements, comparable to signals presented in Fig. 4. However, an exact quantitative comparison is not possible due to the numerous necessary assumptions, in particular on light propagation.

Finally, we cannot completely rule out the possibility of the laser Doppler instrument performing suboptimal either due to poor probe placement, skin compression and/or interference from our NIRS system. Such factors could also explain the rather small task-evoked changes observed in the skin blood flow measurement (see Fig. 6e), compared to our previous studies (Tachtsidis et al., 2009). Summarizing the discussion of the laser Doppler results, we conclude that the relative contributions of the superficial vasculature and big cutaneous veins to the task-evoked systemic signals in fNIRS signals cannot be quantified within the scope of this study.

### Physiological origin

The task-evoked changes in peripheral physiology shown in Fig. 6 provide an insight into possible origins of cutaneous vasodynamics. An increase of skin conductance indicates an increase in sympathetic activity during the CPT-task performance (see Fig. 6c). Increase of the arterial blood pressure can be attributed to an increase of the cardiac output or a task-evoked vasoconstriction in the extremities (see Fig. 6a). The slight decrease in skin blood flow as measured by laser Doppler (see Fig. 6e) indicates that vasoconstriction also occurs in the skin vessels of the forehead.

Skin blood flow and skin blood volume in the forehead are subject to autonomous neural control via three distinct mechanisms: the sympathetic vasoconstrictor, the sympathetic vasodilator and the parasympathetic vasodilator tone. Due to sympathetic vasoconstriction a decrease in skin blood flow was observed during the performance of arithmetic tasks and during mental stress. The decrease of the oxygenated haemoglobin concentration in venous compartments as observed in our experiment can be attributed to a decrease in venous volume induced by a task-evoked decrease in the arterial inflow due to sympathetic vasoconstriction. Since we expect the metabolic demand of the skin not to be influenced by task performance, the amount of deoxygenated haemoglobin should be less effected by the task, which is in agreement with our fNIRS results (see Fig. 4 and Table 1). The time course of the extracranial signal with respect to the activation onset can be potentially explained by a delayed venous response due to high compliance of the veins. This line of reasoning is similar to the models used to explain the venous component of the BOLD signal.

Task-evoked changes in the skin blood volume in the forehead similar to those observed in our study were recently reported by Takahashi et al. (2011). These authors found a significant increase in blood flow and blood volume in the forehead skin during performance of a word fluency task by concurrent fNIRS and laser Doppler flowmetry. In contrast to our findings they observed an increase in blood flow and in blood volume during the task. They attributed these changes to a task-evoked sympathetic vasodilatation. In addition, they report strong habituation of the superficial haemodynamics from the first to the second stimulation block, which we did not observe in our experiments. Indeed a decrease in facial blood flow was observed for periods of mental stress and mental arithmetic (Drummond, 1996). This finding indicates the complexity of the blood flow regulation of the forehead and its possible dependence on the task and experimental design.

An additional contribution to task-evoked haemodynamic changes in the skin blood volume can originate from the blood exchange with the intracranial compartment. The intracranial and extracranial compartments are interconnected by numerous diploic and emissary veins penetrating the skull. Blood vessels in the dura mater are also subjected to the sympathetic vasomotor control (Zenker and Kubik, 1996). We cannot a priori exclude, therefore, an exchange of blood between the extra- and intracranial compartments as a possible contribution to task-evoked signal changes in the scalp. However, since this blood flow is directed from the extracranial towards the intracranial compartment under normal conditions, and since diploic veins have very small diameters in the forehead, it would be unreasonable to assume that such a mechanism could explain several percent venous-volume changes of several percent as observed in our study.

We used task blocks with a period of 65 s. Therefore both, cerebral response and systemic signals observed in fNIRS and fMRI, lie in the range of low frequency oscillations reported in numerous studies (Diedler et al., 2011). These oscillations in the frequency range between 0.1 Hz and 0.01 Hz are the basis of functional connectivity mapping by resting state fMRI. Although several studies demonstrated that functional connectivity mapping might be realized with fNIRS, a strong systemic component in this frequency range was reported (Mesquita et al., 2010). It may well be that the systemic signals observed in our experiments share a common physiological origin with the very low frequency oscillation phenomena previously reported in literature.

Our data show similar artefact patterns, i.e. extracranial signals, for both continuous performance task and working memory task. Since almost any cognitive and emotional processing also induces a specific pattern of autonomic responses we expect corresponding artefacts on the forehead to be present in nearly any fNIRS study. One has to be cautious, therefore, when designing an experimental paradigm for an fNIRS study on the forehead. It might be possible to mitigate task-evoked haemodynamic changes in the skin if the control condition matches the task with respect to the autonomic response. The results performed in our study are valid for healthy adults, and additional experiments are necessary in order to characterize the task-evoked signals in the skin for other age groups such as infants or seniors. But, since the autonomic control is age-dependent and might be systematically changed by certain disorders such as schizophrenia or depression, care has to be taken when performing comparative fNIRS studies.

We observed pronounced artefacts for oxygenated haemoglobin but not for Deoxy-Hb concentration. This may provide a tool to avoid false positives in fNIRS activation maps, i.e. to determine activation maps from the Deoxy-Hb concentration changes only. The analysis of Deoxy-Hb signals in fNIRS is often hampered by their smaller magnitude compared to changes in Oxy-Hb concentration. However, the higher reliability of the Deoxy-Hb activation maps is often discussed in the literature and is also supported by the fact that Deoxy-Hb signals show higher correlations with fMRI results (Cui et al., 2011).

Another way to suppress the superficial artefacts is to include an additional systemic predictor in the GLM analysis of fNIRS data. By using additional predictors derived from a skin fMRI signal we succeeded in separating cerebral and extracranial contributions in the fNIRS signals. However, fMRI information is not available in most of fNIRS studies. Therefore alternative methods to characterize the cutaneous venous contribution have to be used, for example an additional short-separation source-detector pair placed in the vicinity of the vein. Further understanding of the physiological origins and biophysical mechanisms of task-evoked skin haemodynamic responses is desirable in order to generalize the strategy used in our study and to provide a haemodynamic response function of the skin. If a haemodynamic response function of the skin could be determined, this would provide a skin predictor for other tasks and different experimental designs, such as an event related design or block design with different block lengths.

### Cerebral activation

After correcting for superficial signals, activation was observed in fNIRS over bilateral BA10 with a lateralization to the right. This finding is in correspondence with the fMRI data, and can be attributed to the processing of task-related sub-goals (Braver and Bongiolatti, 2002). In our case, it is the semantic categorization on top of the working memory task that has to be managed in the sem-CPT, whereas the word-CPT does not involve an additional semantic task. The average amplitude of the cerebral fMRI signal changes in the activated areas was found to be 0.2%, which is much lower in comparison to signal changes typically observed on 3 T scanners for motor or visual tasks (~ 4%). However, our results demonstrate that when corrected for superficial artefacts, fNIRS is sensitive enough to detect relatively small haemodynamic responses in such complex experimental paradigms.

In addition to the activation in BA 10, we observed a deactivation in the medial regions of the prefrontal cortex in the fMRI experiment. Similar findings have been reported for a variety of cognitive tasks and can be attributed to functions of the default-mode network (Fox and Raichle, 2007). This deactivation was not detectable in our fNIRS data, as the medial region was not covered by a fNIRS channel (see Fig. 2).

### Skin fMRI signals

In addition to improving the interpretability of fNIRS data, we believe that our findings also have an important implication for fMRI studies. The observed task-evoked changes in the fMRI signal of the forehead veins opens up a possibility to detect task-evoked sympathetic responses directly from the conventional whole-brain fMRI images. Currently, an increasing number of fMRI studies on emotional processing use simultaneous measurements of peripheral physiological parameters such as heart rate and SC in order to get independent measures of the bodily arousal (Gray et al., 2009). These measurements require additional MR compatible hardware and additional subject preparation time. We have demonstrated that desirable information is already contained in the extracranial part of the fMRI signals but is currently simply neglected in the fMRI data analysis where a mask is usually applied to exclude all non-brain tissue from the statistical analysis.

### Limitations of the study

Several limitations of the current study need to be considered before general conclusions on the task-evoked artefacts in fNIRS measurements on the forehead can be drawn.

First of all, although our argumentation is based on the comparison of time courses of fMRI and fNIRS signals, these two measurements were not performed simultaneously. We therefore have to critically compare the experimental conditions in the separate fNIRS and fMRI sessions with respect to possible differences in the autonomic states. Three relevant differences were present, (i) the posture of the subjects during the experiment, (ii) differences in the environment comparing the MR scanner and the NIRS lab, (iii) the familiarity of the task during the fMRI session, which was run after the fNIRS session for most of the subjects. The first two factors, posture and environment, are known to influence the general level of the baseline sympathetic output. However, although the rate of spontaneous sympathetic bursts is lower in supine than in sitting position, the sympathetic reaction to the stimuli, which is relevant for task-evoked skin signals, is not influenced by the posture (Boucsein, 2012). The third factor, the task familiarity, can indeed influence the sympathetic response to the task performance. However, our analysis was not based on a quantitative comparison of fMRI and fNIRS signals, but only on the similarity of their time courses. This means that even if the amplitude of the venous response might be different in the fMRI and fNIRS sessions, the time course of the skin haemodynamics is likely to be the same. Thus the GLM analysis of fNIRS signals based on the information from fMRI is justified. The quantitative comparison of fMRI and fNIRS superficial signals might provide important information about task-evoked haemodynamics of the skin and will be a matter for further studies. Such comparison requires not only concurrent fMRI/fNIRS measurements but also more realistic models of light propagation in the head with an anatomically precise representation of the venous compartment.

Furthermore, in our study we used a block design with a fixed duration of task and rest periods. Since the physiological response function of the cutaneous haemodynamics is unknown, additional experiments have to be performed in order to transfer these results to other experimental designs such as event related design and block design with different block length. In particular, future studies have to check to which extent the observed artefacts can be avoided by using block designs with variable block length.

Finally, the amplitude of the cerebral haemodynamic signals due to common activation tasks in the prefrontal cortex seems to be much smaller compared to signals from the primary sensory and motor areas. Due to all these reasons the relative contribution of the task-evoked skin signals measured at the forehead might be notably high.

### Outlook

There are two important implications of our results for those fNIRS studies of the forehead, where correction for the haemodynamics of the superficial layers is employed.

i. The superficial task-evoked artefacts are not homogeneously distributed among the scalp, but are rather localized in the scalp draining veins. This means that the assumption of homogeneous layers cannot be employed when performing multi-distance fNIRS measurements. The relative position of the veins with respect to fNIRS sources and detectors would have to be taken into account.
ii. An important feature of the venous task-evoked signals is its temporal correlation with the haemodynamic cerebral response due to the fact that both of them are task-evoked. In the particular case of our study the correlation coefficient between the predictors modelling extracranial and cerebral signals was found to be 0.39. This fact has to be taken into account when a superficial signal regression is performed based on fNIRS measurements at a short distance. Typically, the short-distance signal is scaled by a factor and subtracted from the long-distance signal. In several studies (Saager and Berger, 2005; Gregg et al., 2010) the scaling factor was obtained by optimisation methods which minimize the temporal correlation between superficial and cerebral components. Even in the case when superficial and cerebral component are correlated the superficial signal regression will force minimal possible correlation. In such case a fraction of the cerebral signal can accidentally be removed.

The interesting question would be if the task-evoked artefacts observed in our study are specific for the forehead or if they also occur in other areas of the scalp. Although task-evoked superficial signals were also reported on the occipital lobe (Boas et al., 2004; Minati et al., 2011) and in the motor area (Franceschini et al., 2006), there might be several reasons why the task-evoked haemodynamics in the forehead skin have stronger effects on fNIRS measurements (Tachtsidis et al., 2009; Takahashi et al., 2011). On the one hand the sympathetic vasoconstriction seems to be more pronounced in the forehead (Drummond, 1996). On the other hand, due to the presence of air filled sinuses the distance from the skin surface to the brain may be larger, thus the sensitivity to the brain activation is lower in this area (Okamoto et al., 2004).

## Conclusions

We have shown that task-evoked haemodynamic changes of veins draining the scalp can induce artefacts in fNIRS signals. We observed strong changes in the tissue concentrations of extracranial Oxy-Hb, but not of Deoxy-Hb. Extracranial and cerebral contributions to the fNIRS signal can be separated by means of a regression analysis. We conclude that a task-evoked decrease in venous volume is present, induced by sympathetic arterial constriction during the task performance. We can estimate the size of this decrease in venous volume from the fMRI signal change. Since changes in sympathetic outflow are concomitant with almost any cognitive and emotional process, we expect similar scalp vessel artefacts as observed in the present study to be present for a wide range of tasks used in neurocognitive research. Caution should be exercised when interpreting such results. Further studies are needed to better characterize the conditions under which superficial systemic artefacts will be substantial and correction is indicated.

The following are the supplementary materials related to this article.

Supplementary material.

## Figures and Tables

**Table 1:** Results of the General Linear Modelling analysis of the fNIRS data. The group averaged amplitudes and corresponding standard-errors-of-mean for Oxy-Hb and Deoxy-Hb concentration changes attributed to cerebral activation (upper part) and extracranial signals (bottom part) are presented in μM. The channels which show significant signals (p < 0.05) are indicated by an asterisk. The time courses of cerebral and extracranial predictors are shown in Figs. 2b and 3d respectively. Corresponding *t*-value activation maps are presented in Fig. 5.

Cerebral activation				
	Channel 1	Channel 2	Channel 3	Channel 4
Δc(Oxy-Hb) (attenuation)	0.065 ± 0.056	− 0.028 ± 0.038	0.12 ± 0.055	0.13 ± 0.05 *
Δc(Deoxy-Hb) (attenuation)	− 0.034 ± 0.02	− 0.038 ± 0.03	− 0.042 ± 0.015 *	− 0.053 ± 0.017 *
Δc(Oxy-Hb) (V)	− 0.034 ± 0.072	− 0.0016 ± 0.0168	0.0259 ± 0.0129	0.04 ± 0.0176
Δc(Deoxy-Hb) (V)	− 0.085 ± 0.04	− 0.016 ± 0.03	− 0.037 ± 0.013 *	− 0.038 ± 0.011 *

*Extracranial signals*				
Δc(Oxy-Hb) (attenuation)	0.112 ± 0.034 *	0.18 ± 0.03 *	0.19 ± 0.05 *	0.084 ± 0.037 *
Δc(Deoxy-Hb) (attenuation)	− 0.002 ± 0.008	− 0.016 ± 0.023	− 0.009 ± 0.011	0.006 ± 0.014
Δc(Oxy-Hb) (V)	0.065 ± 0.026 *	0.097 ± 0.015 *	0.052 ± 0.015 *	− 0.0155 ± 0.0112
Δc(Deoxy-Hb) (V)	0.0099 ± 0.0174	− 0.013 ± 0.087	0.003 ± 0.009	0.009 ± 0.007

## Appendix 1

The concentration changes of Oxy-Hb and Deoxy-Hb were obtained from the light attenuation changes and from the variance of the DTOFs by means of the following procedure. For each wavelength and each channel, we calculated the time course of the integral total photon count *N*_0_ (light intensity), mean time of flight (MTF) and variance *V* of a DTOF. For the calculation of the moments the upper and lower integration limits were fixed to the time points where photon count for a DTOF averaged over the whole time series reached 1% of its maximum. MTF and variance data were corrected for the influence of the instrumental response function (IRF) by subtracting the corresponding moments of the IRF.

### Oxy-Hb and Deoxy-Hb from the light attenuation changes

The attenuation-based concentration changes in Oxy-Hb and Deoxy-Hb were derived from the time courses of the total photon count *N*_0_ of the three wavelengths by using the modified Beer–Lambert law and least square matrix inversion. We used haemoglobin absorption spectra from (Matcher et al., 1995). Differential pathlength factors (DPF) were not taken from tabulated values but calculated from the experimentally measured mean time of flight of photons for each subject, each detection position and each wavelength. The DPF values averaged over all 14 subjects were 6.40 ± 0.20, 5.97 ± 0.16, 5.70 ± 0.18 (mean ± std) for 689 nm, 797 nm and 828 nm, respectively.

### Oxy-Hb and Deoxy-Hb from the variance *V* of photon DTOF

Concentration changes in Oxy-Hb and Deoxy-Hb were also estimated from the time course of the variance *V*. The sensitivity factors for variance (VSF) were determined from the third centralized moment of the DTOF (Liebert et al., 2004), which was obtained from the experimental data for each subject. The VSFs averaged over the subject population were (-1.21 ± 0.16) cm ns^2^, (-1.30 ± 0.10) cm ns^2^ and (-1.3 ± 0.2) cm ns^2^ (mean ± std) for 689 nm, 797 nm and 828 nm, respectively. It should be noted that this approach to calculate the VSF is based on the assumption that the absorption change is homogeneous over the whole volume probed, the same assumption that underlies the DPF concept for the modified Beer–Lambert law for attenuation changes.

## Appendix 2

The amplitude *S* of the fMRI signal averaged over the all voxels containing the cutaneous veins is determined by venous blood volume and venous blood relaxation time:

A1 $$S\sim M_{v}*\exp\left(-\frac{T_{E}}{T_{2,Hb}}\right)$$

where *M*_*v*_ is the total amount of water protons in the vein and 1/*T*_2,Hb_ is transverse relaxation rate resulting from the interaction of water protons with deoxygenated haemoglobin in the blood. *M*_*v*_ is proportional to the total blood volume, and therefore to the total amount of haemoglobin in the veins, so Eq. (A1) can be rewritten as

A2 $$S\sim \left(\text{Σ}\left(\text{OxyHb}\right)+\text{Σ}\left(\text{DeoxyHb}\right)\right)*\exp\left(-\frac{T_{E}}{T_{2,Hb}}\right)$$

where Σ(Oxy-Hb) and Σ(Deoxy-Hb) are total amounts of the venous Oxy-Hb and Deoxy-Hb respectively. It was theoretically (Jensen and Chandra, 2000) and experimentally (Uludag et al., 2009) shown that the relaxation rate of blood water molecules, diffusing in the inhomogeneous field of by red blood cells can be described by the following relationship:

A3 $$\frac{1}{T_{2,Hb}^{}}=\frac{114}{S}*{\left(\frac{\text{Σ}\left(\text{DeoxyHb}\right)}{\text{Σ}\left(\text{DeoxyHb}\right)+\text{Σ}\left(\text{OxyHb}\right)}\right)}^{2}$$

Inserting Eq. (A3) into Eq. (A2) and differentiating it with respect to ΔΣ(Oxy-Hb) and ΔΣ(Deoxy-Hb), the relative amplitude of fMRI signal changes can be calculated:

A4 $$\frac{\text{Δ}S}{S}=\left(\frac{\text{ΔΣ}\left(\text{OxyHb}\right)}{\text{Σ}\left(\text{OxyHb}\right)+\text{Σ}\left(\text{DeoxyHb}\right)}+\frac{\text{ΔΣ}\left(\text{DeoxyHb}\right)}{\text{Σ}\left(\text{OxyHb}\right)+\text{Σ}\left(\text{DeoxyHb}\right)}\right)+\frac{2T_{E}}{T_{2,Hb}}\left(\frac{\text{ΔΣ}\left(\text{OxyHb}\right)}{\text{Σ}\left(\text{OxyHb}\right)+\text{Σ}\left(\text{DeoxyHb}\right)}-\frac{\Sigma \left(\text{OxyHb}\right)}{\Sigma \left(\text{DeoxyHb}\right)}\frac{\text{Δ}\Sigma \left(\text{DeoxyHb}\right)}{\Sigma \left(\text{OxyHb}\right)+\Sigma \left(\text{DeoxyHb}\right)}\right)$$

where the term in the first brackets describes the fMRI signal changes induced by a reduction in the venous volume and the term in the second brackets describes signal changes due to the changes in the relaxation time. Taking the experimentally used value of *T*_E_ = 30 ms and assuming a venous oxygenation level of 70% we can estimate the factors in the Eq. (A4)

A5 $$\frac{\text{Δ}S}{S}=\left(\frac{\text{ΔΣ}\left(\text{OxyHbO}\right)}{\text{Σ}\left(\text{OxyHb}\right)+\text{Σ}\left(\text{DeoxyHb}\right)}+\frac{\text{ΔΣ}\left(\text{DeoxyHb}\right)}{\text{Σ}\left(\left(\text{OxyHb}\right)\right)+\text{Σ}\left(\text{DeoxyHb}\right)}\right)+0.6*\left(\frac{\text{ΔΣ}\left(\left(\text{OxyHb}\right)\right)}{\text{Σ}\left(\text{HbO}\right)+\text{Σ}\left(\text{DeoxyHb}\right)}-2.3\frac{\text{ΔΣ}\left(\text{DeoxyHb}\right)}{\text{Σ}\left(\text{HbO}\right)+\text{Σ}\left(\text{DeoxyHb}\right)}\right)$$

The scaling factors before first and second brackets indicate that the venous volume reduction and enhanced relaxivity due to the increased Deoxy-Hb concentration in the blood contribute with weights of 5/8 and 3/8, respectively, to the signal loss in fMRI.
