# Supplementary material for: The physiological origin of task-evoked systemic artefacts in functional near infrared spectroscopy
Source: Neuroimage. 2012 May 15;61(1):70–81. doi: 10.1016/j.neuroimage.2012.02.074 (PMC3348501; doi:10.1016/j.neuroimage.2012.02.074)
Supplement: Supplementary file 1 — Supplementary material. [file mmc1.doc]

# Supplementary Material

| **Brain Areas** | **Brodmann Areas** | **Cluster Size in Volxes** | | **x** | **y** | **z** | **Max t-Value/Avg t-Valuee** |
| --- | --- | --- | --- | --- | --- | --- | --- |
| **Regions with decreased BOLD signal (mit T-Value 3.23,) (p<0.007143)** | | | | | | | |
| M Default Mode Network | Left Cerebrum, Limbic Lobe, Posterior Cingulate, BA 23 | | 25534 | -2 | -53 | 18 | -16.3/-5.7 |
| M Default Mode Network | Right Cerebrum, Frontal Lobe, Medial Frontal Gyrus, BA 10 | | 17567 | 3 | 56 | 13 | -10.7/-4.5 |
| L Default Mode Network | Left Cerebrum, Temporal Lobe, Middle Temporal Gyrus, BA 39 | | 8098 | -43 | -72 | 19 | -12.8/-4.8 |
| R Default Mode Network | Right Cerebrum, Frontal Lobe, Middle Frontal Gyrus, Gray Matter, BA 10, | | 8680 | 47 | -65 | 15 | -8.6/-4.3 |
| R Default Mode Network, | Right Cerebrum, Limbic Lobe, Parahippocampal Gyrus, Hippocampus | | 1628 | 25 | -11 | -15 | -6.2/-4.0 |
| L Default Mode Network | Left Cerebrum, Limbic Lobe, Parahippocampal Gyrus, Hippocampus | | 1190 | -25 | -12 | -13 | -5.2/-3.8 |
| R Insula | Right Cerebrum, Sub-lobar, Claustrum | | 3526 | 37 | -14 | 10 | -8.9/-4.4 |
| R Right Motor Cortex | Right Cerebrum, Parietal Lobe, Sub-Gyral, GM, BA 40 | | 5392 | 24 | -38 | 54 | -9.9/-4.7 |
| **Regions with increased BOLD signal** | | | | | | | |
| L Motor Cortex M1 | Left Cerebrum, Frontal Lobe, Precentral Gyrus, GM, BA 4 | | 12342 | -37 | -16 | 53 | 11.3/4.8 |
| L SomatoSensory Area SMA | Left Cerebrum, Frontal Lobe, Medial Frontal Gyrus, Gray Matter, BA 6 | | 11274 | -1 | 1 | 55 | 19.6/5.3 |
| R Cerebellum | Right Cerebellum, Anterior Lobe, Culmen, GM | | 7722 | 4 | -52 | -18 | 13.2/5.3 |
| R Primary Visual Cortex V1 | Right Cerebrum, Occipital Lobe, Lingual Gyrus, Gray Matter, BA 17 | | 5116 | 13 | -82 | -18 | 7.2/4.1 |
| L Primary Visual Cortex V1 | Left Cerebrum, Occipital Lobe, Lingual Gyrus, GM, BA 17 | | 7760 | -17 | -97 | -10 | 14.3/5.3 |
| L Fusiform Area | Left Cerebrum, Temporal Lobe, Fusiform Gyrus, GM, BA 37 | | 3585 | -35 | -76 | -18 | 8.1/4.5 |
| L Fusiform Area Visual Word Form Area | Left Cerebrum, Temporal Lobe, Fusiform Gyrus, GM, BA 37 | | 2386 | -42 | -55 | -18 | 7.8/4.4 |
| L Fusiform Area | Left Cerebrum, Temporal Lobe, Fusiform Gyrus, GM, BA 37 | | 2354 | -41 | -41 | -18 | 7.9/4.7 |
| L IPS | Left Cerebrum, Parietal Lobe, Inferior Parietal Lobule, GM, BA 40 | | 8360 | -35 | -52 | 39 | 13.3/4.6 |
| R IPS | Right Cerebrum, Parietal Lobe, Inferior Parietal Lobule, GM, BA 40 | | 7752 | 37 | -51 | 37 | 7.0/4.4 |
| L Left frontal Eye Field | Left Cerebrum, Frontal Lobe, Middle Frontal Gyrus, GM, BA 6 | | 7671 | -42 | 0 | 38 | 11.3/4.9 |
| R Right frontal Eye Field | Right Cerebrum, Frontal Lobe, Precentral Gyrus, GM, BA 6 | | 4876 | 39 | 2 | 35 | 10.3/4.6 |
| L Insula Attention, Motorisch | Left Cerebrum, Sub-lobar, Insula, GM, BA 13 | | 9299 | -37 | 17 | 8 | 14.2/5.2 |
| R Insula Attention | Right Cerebrum, Sub-lobar, Insula, GM, BA 13 | | 7366 | 36 | 19 | 8 | 17.6/5.3 |
| **L sem-CPT+word CPT** | **Left Cerebrum, Frontal Lobe, Middle Frontal Gyrus, GM, BA10** | | **4806** | **-29** | **42** | **14** | **12.3/4.5** |
| **R sem-CPT+word CPT** | **Right Cerebrum, Frontal Lobe, Middle Frontal Gyrus, GM, BA 10** | | **7665** | **32** | **48** | **11** | **9.5/4.5** |
